# Simple Protein Foaming‐Derived 3D Segregated MgO Networks in Epoxy Composites with Outstanding Thermal Conductivity Properties

**DOI:** 10.1002/advs.202506465

**Published:** 2025-05-28

**Authors:** Su‐Jin Ha, Young Kook Moon, Jong‐Jin Choi, Byung‐Dong Hahn, Cheol‐Woo Ahn, Kyung‐Hoon Cho, Hyun‐Ae Cha

**Affiliations:** ^1^ Nano Materials Research Division Korea Institute of Materials Science (KIMS) Changwon Gyeongnam 641‐831 Republic of Korea; ^2^ School of Materials Science and Engineering Kumoh National Institute of Technology 61 Daehak‐ro Gumi Gyeongbuk 39177 Republic of Korea

**Keywords:** 3D segregated structure, MgO thermal filler, polymer–matrix composites, protein foaming methods, thermal conductivity

## Abstract

The miniaturization and high‐power density of electronic devices present new challenges for thermal management. Efficient heat dissipation in electrically insulating packaging materials is currently limited by the thermal conductivity of thermal‐interface materials (TIMs) and their ability to effectively direct heat toward heat sinks. In this study, MgO‐based composites with high thermal conductivities are fabricated to achieve excellent thermal performances by optimizing the heat‐transfer path. These composites are produced using a protein foaming method, which effectively forms interconnected ceramic‐filler networks. Additionally, the liquid phase formed during the sintering of MgO enhances the bonding with the epoxy matrix, thereby improving the thermal conductivity of the composites. As a result, the composites with 54.64 vol% MgO achieve a high thermal conductivity of 17.19 W m^−1^ K^−1^, which is 101 times higher than that of pure epoxy, 3.7 times higher than that of randomly dispersed composites, and even superior to that of nitride‐based composites. Moreover, the composites also exhibited a low thermal‐expansion coefficient (27.76 ppm °C^−1^) and high electrical‐insulation strength (51.51 kV mm^−1^), ensuring good thermal and electrical performance for electronic‐packaging applications. The strategic design of the TIM microstructures for effectively directing heat offers a promising solution for efficient thermal management in integrated electronics.

## Graphical abstract

MgO‐based composites with highly interconnected filler networks are fabricated using a simple, eco‐friendly protein foaming method. Liquid‐phase‐assisted sintering enhanced bonding with the matrix. The interconnected structured filler composites exhibit significantly improved thermal conductivity, exceeding that of pure epoxy, randomly dispersed composites, and even nitride‐based systems, offering an effective strategy for next‐generation thermal management.

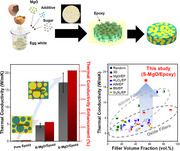

## 1 Introduction

As electronic devices become increasingly multifunctional with advancements in integration and miniaturization, the efficiency of heat‐dissipation packages plays a critical role in ensuring the lifetime and reliability of these devices.^[^ ^1^ ^]^ For example, frequent battery explosions in electric vehicles highlight the urgent need for high‐performance thermal‐management systems, especially because future electric vehicles will require high‐power, high‐speed charging and discharging capabilities. Therefore, next‐generation thermal‐management solutions must be developed to address these demands. Thermal interface materials (TIMs) are essential components in heat‐dissipation systems and are placed at the contact interface between heat sources and heat sinks to improve the thermal transfer efficiency. When two solid surfaces come into contact, the voids between them are filled with air, which has low thermal conductivity (0.026 W m^−1^ K^−1^), thereby impeding heat transfer. TIMs, which are often made of composite materials with high thermal conductivity, are used as pads or gap fillers to minimize the thermal resistance at these interfaces. Because polymer matrices inherently have a low thermal conductivity (≈0.2 W m^−1^ K^−1^),^[^ ^2^ ^]^ most of the heat transfer in these materials occurs through the incorporated fillers, making the thermal conductivity of the filler critical to the overall performance of the TIM.

Ceramic fillers such as alumina (Al_2_O_3_),^[^ ^3^, ^4^, ^5^, ^6^, ^7^, ^8^, ^9^ ^]^ alumina nitride (AlN),^[^ ^10^, ^11^ ^]^ boron nitride (BN),^[^ ^12^, ^13^, ^14^ ^]^ and silicon nitride (Si_3_N_4_)^[^ ^15^, ^16^, ^17^, ^18^ ^]^ are widely utilized in applications requiring electrical insulation. Among these, alumina is the most prevalent, constituting ≈98% of thermal ceramic fillers owing to its cost‐effectiveness. However, the thermal conductivity of alumina, ranging from 20 to 30 W m^−1^ K^−1^,^[^ ^3^, ^10^ ^]^ is relatively limited. In comparison, nitrides such as AlN, BN, and Si_3_N_4_ offer superior thermal conductivities exceeding 70 W m^−1^ K^−1^.^[^ ^12^, ^16^ ^]^ Despite their excellent thermal properties, the high processing costs of nitrides significantly restrict their feasibility as replacements for alumina in large‐scale applications. On the other hand, magnesium oxide (MgO) (theoretical density: 3.58 g cm^−3^) offers significant advantages over Al_2_O_3_ (theoretical density: 3.99 g cm^−3^) as it is lighter and has nearly twice the thermal conductivity.^[^ ^19^, ^20^, ^21^, ^22^ ^]^ However, while these benefits are significant, MgO has a high melting point, requires sintering temperatures above 1700 °C, and is susceptible to hydration reactions under ambient conditions. To overcome these problems, a previous study proposed a method for inducing liquid‐phase sintering by adding small amounts of TiO_2_ and Nb_2_O_5_ as sintering additives.^[^ ^20^ ^]^ This approach addressed the high melting point and hydration issues while maintaining a cost comparable to that of Al_2_O_3_. In addition, MgO achieved a smooth surface after sintering because the liquid phase covered the surface of MgO during the sintering process, which helped reduce the surface resistance and improve mixing with the polymer. Unlike traditional chemical‐modification processes, which are often complex and inefficient and may compromise the inherent thermal conductivity of fillers, liquid‐phase sintering provides a simpler and more effective alternative for improving the compatibility between the fillers and matrix. By utilizing a liquid‐phase sintering approach, fillers can achieve a smooth surface without extensive chemical treatment, which significantly reduces the risk of altering their intrinsic thermal conductivities. This smooth surface not only minimizes interfacial resistance, but also reduces phonon scattering at the interface, improves particle‐to‐particle and particle‐to‐matrix connectivity, and directly enhances the structural integrity of the filler network, unlike traditional surface‐functionalization methods that rely on covalent bonds to mitigate phonon mismatch. Unlike chemical modifications that fail to address the fundamental phonon‐spectrum mismatch between the filler and matrix,^[^ ^23^, ^24^ ^]^ liquid‐phase sintering improves thermal transport by creating more continuous and less resistive pathways for heat flow. Consequently, MgO with a smooth surface maintained a higher thermal conductivity than pure MgO, demonstrating the effectiveness of the liquid‐phase sintering method.

When simply compounding fillers with polymers, the thermal conductivity of TIMs increases with filler loading owing to the formation of a percolation network. However, high filler content can adversely affect the adhesion, flexibility, and processability of the material. Therefore, strategies to achieve high thermal conductivity at a low filler content are crucial.^[^ ^25^ ^]^ Arranging ceramic fillers within a polymer matrix in an aligned or 3D configuration can significantly enhance thermal properties compared with randomly dispersed composites. The heat‐transfer efficiency was improved by strategically organizing the fillers to create efficient thermal pathways, allowing for high thermal conductivity even at lower filler concentrations. Various techniques have been developed to construct segregated structures with anisotropic/3D filler arrangements. The fabrication of segregated ceramic structures and filler alignment is achieved using techniques such as gel casting,^[^ ^26^ ^]^ direct foaming,^[^ ^27^, ^28^ ^]^ pore foaming,^[^ ^29^, ^30^, ^31^ ^]^ sacrificial templates,^[^ ^32^, ^33^, ^34^, ^35^, ^36^, ^37^, ^38^ ^]^ hot pressing,^[^ ^39^, ^40^ ^]^ magnetic fields,^[^ ^41^, ^42^, ^43^ ^]^ filtration methods,^[^ ^44^, ^45^ ^]^ and electrospinning.^[^ ^46^, ^47^ ^]^ These techniques enable the formation of continuous filler networks, thereby enhancing the thermal conductivity of the composites while maintaining excellent mechanical properties and processability. However, although hot pressing, magnetic fields, filtration methods, and electrospinning are effective in aligning fillers, they encounter significant challenges when constructing complex 3D networks. These techniques primarily result in layered or directionally constrained structures, which limit the formation of fully interconnected heat‐conduction pathways.^[^ ^39^, ^40^, ^41^, ^42^, ^43^, ^44^, ^45^, ^46^, ^47^ ^]^ In contrast, techniques such as gel casting, direct foaming, pore foaming, and sacrificial templates have been widely employed in studies requiring 3D‐network formation because they enable the construction of highly interconnected and continuous ceramic frameworks.^[^ ^26^, ^27^, ^28^, ^29^, ^30^, ^31^, ^32^, ^33^, ^34^, ^35^, ^36^, ^37^, ^38^ ^]^ These methods facilitate the creation of percolated thermal pathways by promoting a uniform filler distribution and network connectivity, making them particularly suitable for applications that require efficient heat transfer. A previous study demonstrated that gel casting could achieve up to 70 vol% Al_2_O_3_ content, enhancing thermal conductivity up to 13.46 W m^−1^ K^−1^.^[^ ^26^ ^]^ Gel casting utilizes high‐solid‐content slurries to uniformly disperse ceramic particles and form interconnected networks, thereby effectively enhancing the thermal conductivity. This level of thermal conductivity is considered exceptionally high for oxide‐based ceramics. As demonstrated in a previous study, direct‐forming methods enabled the fabrication of Al_2_O_3_/epoxy composites with a continuous 3D alumina framework.^[^ ^2^ ^]^ This approach, combined with high‐temperature sintering, reduced the interfacial thermal resistance and enhanced the mechanical strength, achieving a thermal conductivity 3.6 times higher than that of the composites with randomly dispersed Al_2_O_3_ at the same filler loading.

The protein foaming method used in this study can be regarded as a combination of the principles of gel casting and direct forming, enabling the formation of highly interconnected 3D ceramic networks with a high packing density. During this process, proteins function as both foaming agents and binders. Their amphiphilic properties allow them to stabilize the air–water interface, resulting in uniform bubbles encapsulating the ceramic fillers. This approach enables the homogeneous dispersion of ceramic fillers within the slurry and facilitates the formation of self‐organized, dense, and continuous networks through phase separation and capillary‐driven assembly during solidification. Moreover, the high solid‐loading capability of this method ensures that a large volume fraction of fillers is homogeneously distributed without significant aggregation, thereby forming well‐aligned thermal‐conduction networks that minimize thermal resistance and enhance heat‐transfer pathways. The protein foaming method enables densely interconnected filler networks and efficient thermal pathways compared to the sacrificial‐template method, which often results in loose packing and poor interconnectivity owing to limitations in dynamic particle redistribution. Furthermore, because this method utilizes natural and renewable resources, such as egg albumen,^[^ ^2^, ^48^, ^49^, ^50^, ^51^, ^52^ ^]^ and avoids toxic or complex chemicals,^[^ ^37^, ^38^ ^]^ it provides an environmentally friendly and scalable approach for fabricating 3D segregated porous networks in ceramic or polymer composites.

## 2 Results and Discussion

### 2.1 Design Conception and Preparation of S‐MgO/Epoxy Composites

**Figure 1:**
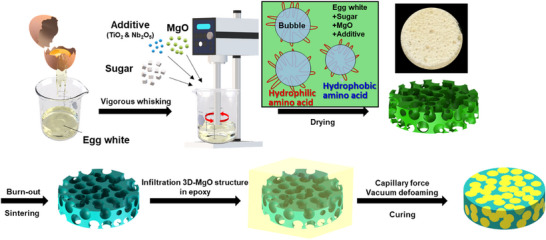
Schematic illustrations of the fabrication process of simple protein foaming derived S‐MgO/epoxy composites.

Proteins derived from egg albumen and sugar powder were used as precursors to fabricate freestanding 3D segregated MgO(S‐MgO) structures. **Figure** **1** shows a schematic illustration of the fabrication process for the protein foaming‐derived S‐MgO/epoxy composites. Egg albumen consists of 90% water and 10% protein. During vigorous whisking, the proteins undergo denaturation, unfolding into amino acid chains. The hydrophilic segments of the unfolded proteins interact with water molecules, whereas the hydrophobic segments interact with air bubbles, forming a gel‐like interfacial membrane. This membrane stabilizes air bubbles within the protein network and plays a crucial role in maintaining the foam structure. When proteins and sugars interact, they undergo the maillard reaction, where sugar molecules, such as sucrose, react with the amino groups (─NH₂) of proteins, leading to cross‐linking. This cross‐linking connects the protein molecules, resulting in a more rigid and structured network. Therefore, sugar enhances the rigidity of the protein walls, ensuring the stability of the porous structure, even during the baking process.^[^ ^48^ ^]^ To verify the stability of this foam structure, a meringue network was fabricated using pure albumen without MgO, and as shown in Figure S1 (Supporting Information), the protein network stabilized by sugar exhibited a porous sponge‐like morphology. This confirms that the protein network can form and maintain stable pores, providing a structural foundation for the subsequent incorporation and uniform dispersion of MgO and enabling its conversion into an inorganic network. Furthermore, the burnout process of the protein was conducted at 500 °C for 2 h, which was sufficient for the complete removal of albumen, as confirmed by the derivative thermogravimetry results in Figure S2 (Supporting Information).

The sintering temperature for the fabricated MgO framework was selected based on the scanning electron microscopy (SEM) images shown in Figure S3 (Supporting Information). Figure S3a (Supporting Information) presents the microstructural evolution of pure MgO with a sintering temperature increasing from 1500 to 1700 °C, and Figure S3b (Supporting Information) illustrates the microstructural changes in MgO containing TiO_2_ and Nb_2_O_5_ sintering additives in the temperature range of 1000–1500 °C. In pure MgO, significant grain growth exceeding 5 µm was observed at ≈1600 °C. However, with the incorporation of sintering additives, grain growth was evident even at lower temperatures, such as 1200 °C, indicating a reduction in the sintering‐onset temperature. Furthermore, Figure S3c (Supporting Information) shows an enhancement of the thermal properties of the sintered MgO upon the addition of sintering additives as the temperature increases. A relative density of 95.3% was achieved at 1200 °C along with grain growth, and at 1400 °C, the maximum density (99.6%) and thermal diffusivity (17.03 mm^2^ s^−1^) were attained. These results indicate that densification is significantly enhanced in the temperature range of 1200–1400 °C, supporting the selection of this range for the sintering process.

### 2.2 Microstructure and Surface Morphology of the S‐MgO Framework

**Figure 2:**
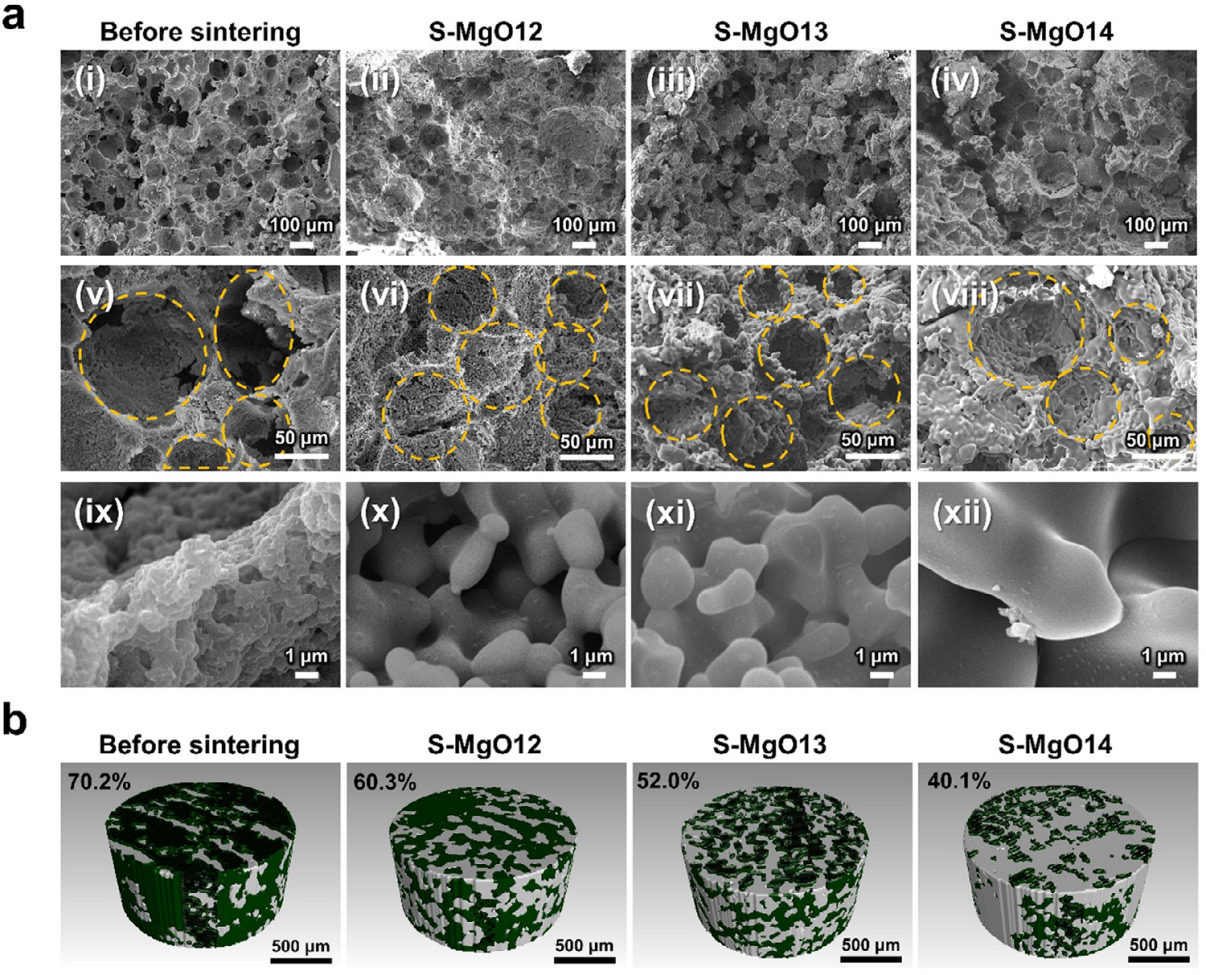
a) SEM images of of S‐MgO framework before sintering, S‐MgO12, S‐MgO13, and S‐MgO14. b) Micro‐CT 3D images of S‐MgO framework before sintering, S‐MgO12, S‐MgO13, and S‐MgO14.

X‐ray diffraction (XRD) patterns of the S‐MgO framework before and after sintering are presented. The S‐MgO framework before sintering exhibited both amorphous and MgO phases (Figure S4, Supporting Information). However, the S‐MgO frameworks sintered at 1200 °C (S‐MgO12), 1300 °C (S‐MgO13), and 1400 °C (S‐MgO14) showed the disappearance of amorphous protein peaks, leaving only well‐defined MgO patterns. This indicates that the albumen phase was completely burned out during the sintering process, resulting in the formation of a pure MgO framework while retaining the overall network structure. SEM images of the MgO/protein green body before sintering and the porous S‐MgO framework for S‐MgO12, S‐MgO13, and S‐MgO14 are shown in **Figure** **2**a. Figure 2a(i–viii) shows that MgO powder with sintering additives is uniformly distributed within the sponge‐like structure of the protein during the foaming process. Even after sintering, the porous network structure remained intact, forming a densely packed and continuous MgO network, as confirmed in Figure S5 (1100–1150 °C, 2 h, Supporting Information) and Figure 2 (1200–1400 °C, 2 h). The microstructure clearly shows that significant grain growth did not occur at 1100 °C. However, from 1150 °C, the MgO particles began to gradually connect (Figure S5, Supporting Information), and at 1400 °C, the interconnectivity was significantly enhanced (Figure 2a). As the sintering temperature increased, the grain size increased significantly. Particularly, the grains exhibited substantial growth in S‐MgO14. The microstructure revealed that the grains grew to such an extent that virtually no gaps were observed between them, resulting in a densely packed configuration. As shown in the X‐ray micro‐computed tomography (micro‐CT) results in Figure 2b, the sintered S‐MgO frameworks exhibited high porosity. With increasing sintering temperature, the porosity decreased significantly, ranging from ≈70.2% before sintering to 40.1% for S‐MgO14. This can be attributed to the enhanced filler networking resulting from the grain growth and thickening of the 3D‐framework walls. The porosity‐analysis results measured by mercury porosimetry showed a trend consistent with that of micro‐CT (Figure S6, Supporting Information). Based on the porosity‐analysis results, the porosity decreased from 57.45% to 45.94% as the sintering temperature increased from 1200 to 1400 °C. This reduction in porosity was attributed to the integration of interfacial layers between the MgO grain boundaries, as observed in the enlarged images (viii–xi) in Figure 2a. Additionally, when the sintering temperature increased from 1300 to 1400 °C, the porosity measured by mercury porosimetry decreased by ≈5% (from 50.54% to 45.94%), whereas the micro‐CT analysis revealed a larger reduction of ≈12% (from 52.0% to 40.1%). This suggests that closed pores increased at 1400 °C. 3D reconstruction confirmed that after sintering, the MgO framework maintained a continuous and well‐interconnected network along all directions (*x*, *y*, and *z*) without any discontinuity in the network, as shown in Figure S7 of the Supporting Information. These results verified that the structural connectivity of the MgO framework was preserved during the sintering process and demonstrated that the protein foaming method effectively formed highly interconnected 3D MgO networks.

**Figure 3:**
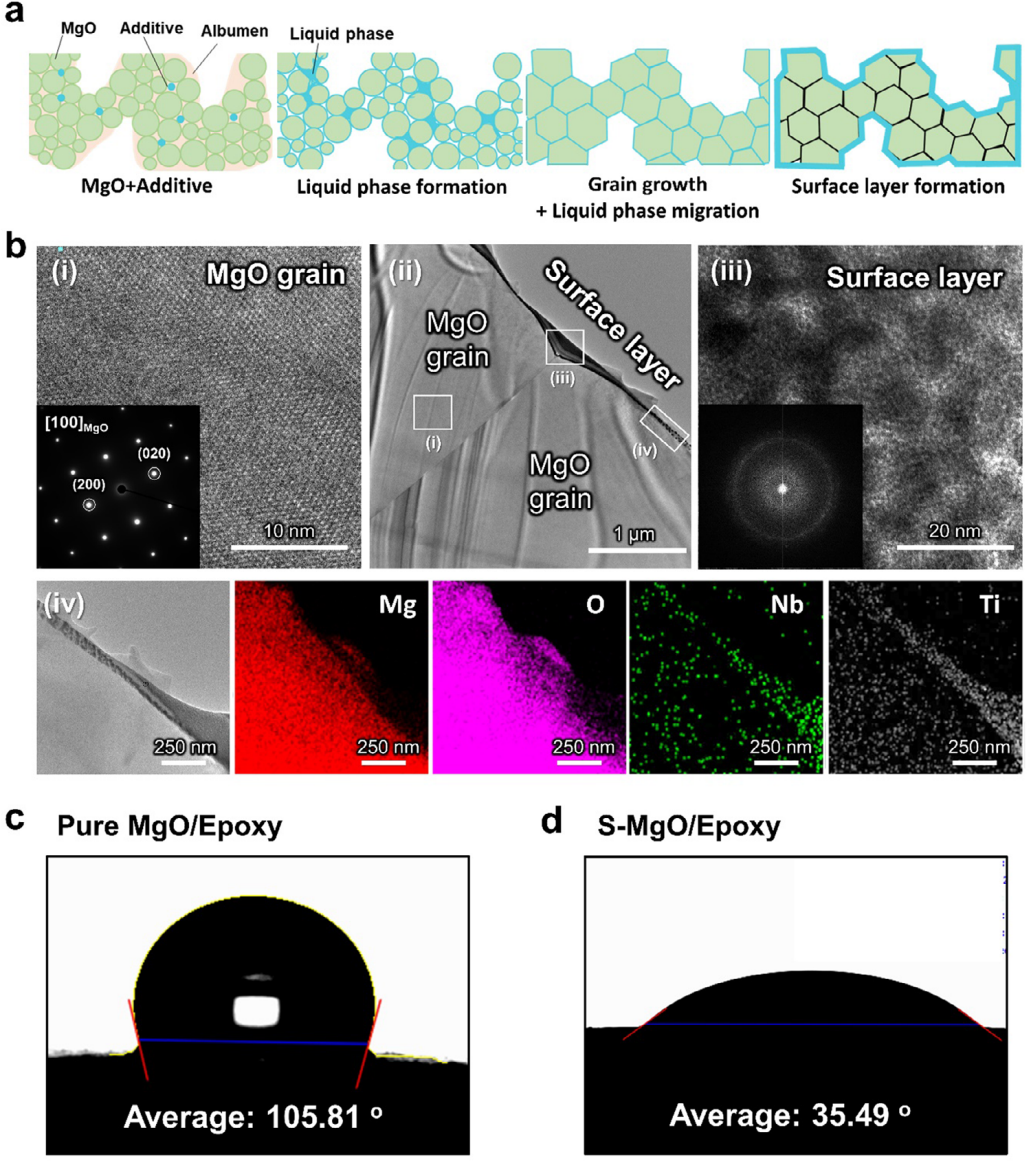
Schematic illustrations of a) the sintering mechanism of S‐MgO during liquid phase sintering using sintering additives. b) TEM image of MgO with additives. Epoxy contact angle with pelleted c) pure MgO and d) MgO with additives.

As shown in **Figure** **3**a, the integration of interfacial layers and increase in closed pores can be attributed to the formation of a liquid phase during sintering induced by the addition of sintering additives. The liquid phase promoted grain growth, and the growing grains became interconnected, leading to the integration of grain boundaries and development of a MgO network. Furthermore, the liquid phase migrated to the particle surface during grain growth, resulting in the formation of a smooth surface layer. The formation of the surface layer can be observed in Figure 2a(vii) and is further supported by Figure S8 (Supporting Information). While pure MgO exhibited a faceted structure with a rough surface, the sample with sintering additives exhibited a significantly smooth surface layer. To confirm this, transmission electron microscopy (TEM) analysis was performed and the results are shown in Figure 3b. Consequently, a surface layer with a thickness ranging from several tens to hundreds of nanometers was observed on the surfaces of the MgO particles. Figure 3b(ii) shows the TEM image of the surface layer formed on the MgO particles. The interior of the MgO grains exhibited a distinct [100] lattice pattern, as shown in Figure 3b(i), whereas the surface layer clearly displayed an amorphous phase in Figure 3b(iii). The amorphous phase, which is shown in Figure 3b(iii), is challenging to detect using XRD in Figure S4 of the Supporting Information owing to its low amount of 0.1–0.15 at%. Notably, Ti and Nb were detected at significantly higher concentrations on the surface layer than in the MgO grains, as confirmed by energy‐dispersive X‐ray spectroscopy (EDS) (Figure 3b(iv); Figure S9, Supporting Information). These observations confirmed that the liquid phase formed during the sintering process migrated to the surface, resulting in the formation of the surface layer. To further investigate the compatibility of the smooth surface layer with epoxy, wettability tests were performed on the sintered dense pellets. Specifically, contact angles were measured between epoxy and pure MgO sintered at 1700 °C, as well as MgO with sintering additives sintered at 1300 °C (Figure 3c,d). The contact angle of pure MgO was 105.81°, but decreased significantly to 35.49° for MgO with sintering additives. This considerable reduction in the contact angle indicates improved wettability with epoxy, suggesting that the smooth surface layer formed during the sintering process contributed to the hydrophobicity of the MgO surface.

### 2.3 Microstructure of the S‐MgO/Epoxy Composites

**Figure 4:**
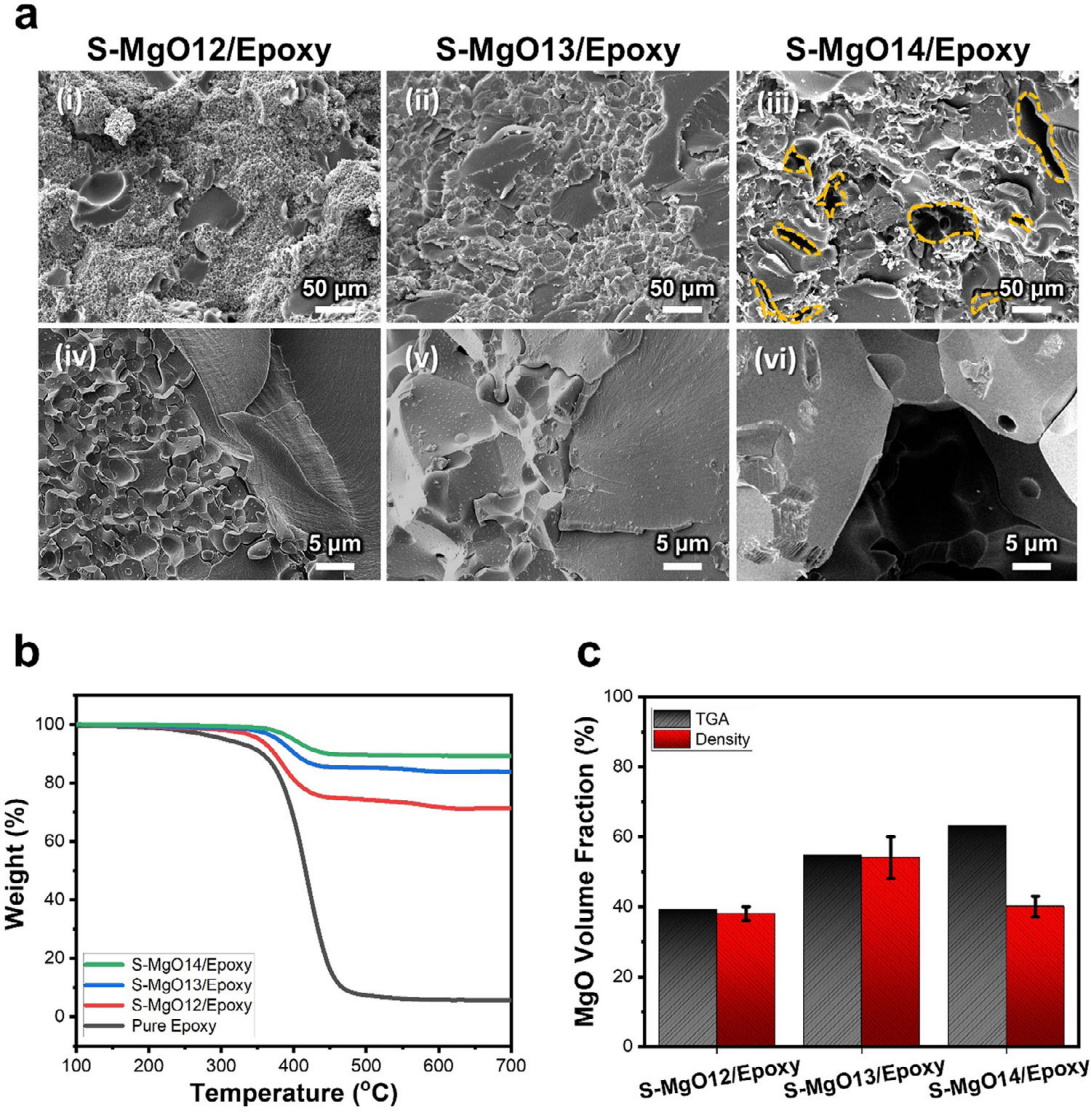
a) Cross‐sectional SEM images of the S‐MgO/epoxy composites under different sintering temperature conditions. b) TGA thermograms of the S‐MgO/epoxy composites under different sintering temperature conditions. c) The MgO volume fraction of the S‐MgO/epoxy composites under different sintering temperature conditions from the calculation of TGA (b) and density.

The fracture surfaces of the composites fabricated under different sintering conditions were examined using SEM to assess the extent of epoxy infiltration, and the results are presented in **Figure** **4**a. The fracture surfaces after epoxy infiltration and curing of the composites at each condition clearly show that epoxy successfully filled S‐MgO when it was sintered at 1200 °C (S‐MgO12/epoxy) and 1300 °C (S‐MgO13/epoxy), as shown in Figure 4a(i,ii). To confirm the infiltration of epoxy, the XRD patterns of pure epoxy and the sintered S‐MgO/epoxy composites are shown for each sintering condition. The epoxy exhibited an amorphous peak in the range of 10°–30°, and a similar amorphous peak was observed in the 10°–30° range for the S‐MgO/epoxy composites (Figure S10, Supporting Information). The results revealed that the composite exhibited an amorphous epoxy phase, which was attributed to the infiltration of epoxy into the MgO network. As shown in Figure S11 (Supporting Information), the EDS elemental‐mapping analysis confirmed that the epoxy infiltrated the MgO network well. Furthermore, the elemental distribution of Mg was continuously observed throughout the entire framework without interruption, indicating that the MgO network was structurally well connected. This result provides additional evidence for the interconnected nature of the S‐MgO structure formed by the protein foaming method. Additionally, as the sintering temperature increased, a higher proportion of MgO relative to epoxy was observed in Figure 4a(iv–vi). This can be interpreted as the result of grain growth during the sintering process, leading to an increase in the MgO filler content. Specifically, the formation of pores in the S‐MgO14/epoxy composite shown in Figure 4a(vi) is likely due to the thickening of the MgO skeleton with increasing sintering temperature, which restricts the effective infiltration of epoxy. The measurement of the filler content in the S‐MgO/epoxy composites was conducted using thermogravimetric analysis (TGA) (Figure 4b). The degradation of the composites was observed between 350 and 460 °C, corresponding to the degradation of the cured epoxy. Thus, the filler volume fractions of the S‐MgO/epoxy composites in S‐MgO12/epoxy, S‐MgO13/epoxy, and S‐MgO14/epoxy composites were 39.15%, 54.64%, and 63.15%, respectively, as shown in Figure 4c. In addition, the filler content measured from the density of the composite material was consistent with the TGA results for the S‐MgO12/epoxy and S‐MgO13/epoxy composites. As the sintering temperature increased, the filler content measured by TGA gradually increased, which could be attributed to grain growth. However, a discrepancy was observed for the S‐MgO14/epoxy composite, owing to the presence of pores. TGA revealed a higher filler volume fraction than the filler content measured using the density of the composite material. In the S‐MgO14/epoxy composite, the incompletely infiltrated epoxy led to the formation of pores, which consequently lowered the overall density. Consequently, the filler content measured from the density appeared to be relatively low.

### 2.4 Enhanced Thermal Conductivity of S‐MgO/Epoxy Composites

**Figure 5:**
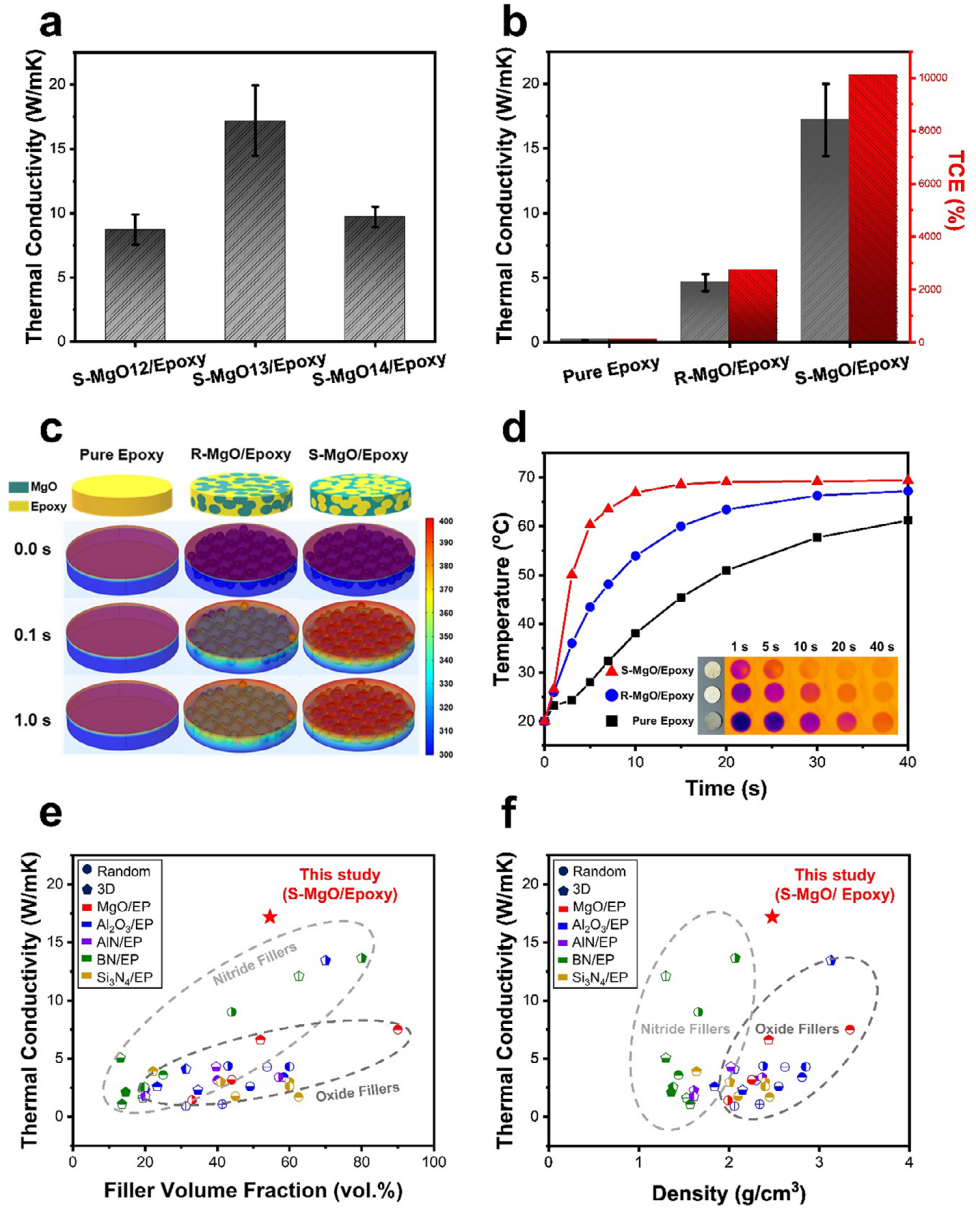
a) Thermal conductivity of the S‐MgO/epoxy composites under different sintering temperature conditions. b) Thermal conductivity and c) COMSOL Multiphysics simulation of pure epoxy, R‐MgO/epoxy composites, and S‐MgO/epoxy composites. d) Thermal management performance of each composite, with infrared thermal images of the samples shown in the inset. Comparison of thermal conductivity results of the S‐MgO/epoxy composite in this study with randomly dispersed (MgO, Al_2_O_3_, AlN, BN, Si_3_N_4_) and 3D‐structured (3D‐MgO, 3D‐Al_2_O_3_, 3D‐AlN, 3D‐BN, 3D‐Si_3_N_4_) filler/epoxy composites from other studies as a function of e) filler volume fraction and f) composite density.

**Figure** **5**a shows the dependence of the thermal conductivity on the sintering temperature. The thermal conductivity of the S‐MgO12/epoxy and S‐MgO13/epoxy composites were 8.73 and 17.19 W m^−1^ K^−1^, respectively. This improvement can be attributed to the reinforcement of the MgO framework network with increasing filler volume fraction because grain growth occurs with increasing sintering temperature, contributing to the formation of more efficient thermal‐transport pathways within the composite. The thermal conductivity of all S‐MgO/epoxy composites was measured using the laser flash analysis (LFA) method. To compare the thermal performance using another evaluation method, the ASTM D 5470 method, which is commonly used in industrial applications, was also applied. Using this method, the S‐MgO13/epoxy composite exhibited a thermal conductivity of 15.43 W m^−1^ K^−1^ (Figure S12, Supporting Information). This value is reasonably consistent with the LFA result (17.19 W m^−1^ K^−1^), indicating that the composite maintains a high thermal conductivity under different evaluation methods. Although the S‐MgO13/epoxy composite maintained a high thermal conductivity under various evaluation methods, the thermal conductivity of the S‐MgO14/epoxy composite decreased to 9.70 W m^−1^ K^−1^. This decrease in thermal conductivity was attributed to the presence of pores in the S‐MgO14/epoxy composite, as shown in Figure 4a. These pores, filled with air with low thermal conductivity (0.0026 W m^−1^ K^−1^), disrupted the continuity of the thermal‐transport pathways and increased the interfacial thermal resistance, thereby significantly reducing the overall thermal conductivity. The influence of pores on thermal‐conductivity degradation is more clearly observed in Figure S13 of the Supporting Information. The S‐MgO15 sample sintered at 1500 °C exhibited pores of a reduced number and size due to densification. However, in the S‐MgO15/epoxy composite, the epoxy infiltration into the internal pores was limited because of the increased skeleton thickness. Consequently, the composite exhibited a relatively low thermal conductivity of 6.46 W m^−1^ K^−1^.

To clearly evaluate the effect of the 3D network structure under identical filler loading conditions, the S‐MgO/epoxy composite was compared with the randomly dispersed MgO(R‐MgO)/epoxy composite. As shown in Figure 5b, the S‐MgO/epoxy composite exhibits significantly enhanced heat‐transfer efficiency compared to pure epoxy and the R‐MgO/epoxy composite. Specifically, the thermal conductivity of the S‐MgO/epoxy composite (17.19 W m^−1^ K^−1^) was significantly higher than that of the R‐MgO/epoxy composite (4.63 W m^−1^ K^−1^). Furthermore, when compared to pure epoxy (0.17 W m^−1^ K^−1^), the S‐MgO/epoxy composite exhibited a thermal‐conductivity enhancement (TCE) of ≈10 000%. These findings confirmed that the filler‐network structure critically influenced the overall thermal‐transport performance of the composite. To further validate this, a finite‐element simulation was conducted using COMSOL Multiphysics 5.3, as illustrated in Figure 5c and Movie S1 (Supporting Information). The heat‐transfer efficiency was analyzed for the R‐MgO/epoxy and S‐MgO/epoxy composites, both containing 50 vol% MgO, and the results showed that the formation of a 3D segregated network significantly improved the heat‐transfer efficiency. This improvement was attributed to the establishment of continuous MgO networks, which optimized the thermal‐transport pathways. Figure S14 (Supporting Information) shows that the difference in filler arrangement between the R‐MgO and S‐MgO composites led to variations in the efficiency of the thermal‐transport pathways. The R‐MgO composite exhibited a discontinuous MgO filler arrangement, which led to an ineffective thermal‐transport structure. Owing to the insufficient connectivity between the fillers, a portion of the heat was conducted through the epoxy matrix, which has a significantly lower thermal conductivity. This structural limitation increased the interfacial resistance, disrupted thermal‐transport pathways, and ultimately increased the overall thermal resistance, resulting in reduced thermal conductivity. In contrast, the S‐MgO composite formed a 3D interconnected network of highly thermally conductive MgO fillers, thereby securing continuous heat‐transfer pathways. This network structure not only facilitated uninterrupted thermal transport, but also reduced the interfacial thermal resistance between the MgO particles. As the direct contact area between the MgO fillers increased, the heat‐transfer efficiency at the interfaces improved, minimizing heat conduction through the epoxy matrix and further lowering the thermal resistance. As a result, the S‐MgO/epoxy composite showed substantially reduced interfacial thermal resistance and greatly enhanced thermal conductivity compared to the R‐MgO/epoxy composite.

The thermal‐transport properties and heat‐dissipation performance of the samples were evaluated using the surface temperatures recorded by an infrared thermal‐imaging camera. For comparison, pure epoxy and the R‐MgO/epoxy composites were also analyzed. The samples were mounted on a ceramic heater of a hot plate, and an infrared thermal imager was used to capture and record the surface‐temperature distribution and variation. Figure S15 of the Supporting Information presents a schematic illustration of the experimental setup, showing the relative positions of the infrared camera, composite sample, and heating stage. The infrared images and time–temperature curves showed that the times required for the surface temperature to reach 60 °C were ≈5, 15, and 40 s for the S‐MgO/epoxy composite, R‐MgO/epoxy composite, and pure epoxy, respectively (Figure 5d). This indicates that the S‐MgO/epoxy composite exhibits the fastest heat‐transfer rate, demonstrating superior thermal conductivity. A series of infrared thermograms is displayed in the inset of Figure 5d. The higher the temperature, the lighter the surface color. Evidently, the surface color of the S‐MgO/epoxy composite appears brighter than those of pure epoxy and R‐MgO/epoxy after 40 s of heating, indicating a faster rate of warming, which demonstrates the significantly enhanced heat‐transfer capacity of the S‐MgO/epoxy composite.

Figure 5e,f shows thermal conductivity comparisons with previously reported ceramic filler/epoxy composites, with detailed information provided in Table S1 of the Supporting Information, where the *x*‐axis represents either the filler volume fraction or density.^[^ ^2^, ^3^, ^4^, ^5^, ^6^, ^7^, ^8^, ^9^, ^10^, ^11^, ^12^, ^13^, ^14^, ^15^, ^16^, ^17^, ^18^, ^20^, ^21^, ^22^, ^26^, ^27^, ^28^, ^29^, ^30^, ^31^, ^32^, ^33^, ^34^, ^35^, ^36^, ^37^, ^41^, ^42^, ^43^, ^52^ ^]^ Despite using a lower filler volume compared to the reference, the composite achieved significantly higher thermal conductivity. Furthermore, the composites exhibited superior thermal conductivity compared to 3D‐Al_2_O_3_^[^ ^2^, ^26^, ^29^, ^41^ ^]^ and nitride‐based 3D composites such as 3D‐AlN,^[^ ^30^, ^31^, ^42^ ^]^ 3D‐BN,^[^ ^27^, ^32^, ^33^, ^34^, ^35^, ^36^, ^43^ ^]^ and 3D‐Si_3_N_4_.^[^ ^28^, ^52^ ^]^ These results emphasized the effect of enhancing the thermal conductivity of highly interconnected networks and improving the interfacial interaction between S‐MgO and epoxy in the developed composites.

The S‐MgO/epoxy composite (17.19 W m^−1^ K^−1^) with 54.64 vol% filler content achieved a higher thermal conductivity than that of the 3D‐Al_2_O_3_/epoxy composite with 70 vol% filler content (13.46 W m^−1^ K^−1^),^[^ ^26^ ^]^ despite having a lower filler volume fraction. These results can be attributed to the inherent difference in thermal conductivity between Al_2_O_3_ and MgO, making MgO‐based composites promising materials that effectively address the requirements of both thermal conductivity and cost‐effectiveness. In particular, MgO, which has a lower density than Al_2_O_3_, provides the additional advantage of high thermal conductivity relative to its density, compared to other thermally conductive ceramic materials. Even when considering the lighter density of nitride‐based materials compared to MgO (AlN: 3.26 g cm^−3^, BN: 2.1 g cm^−3^, Si_3_N_4_: 3.17 g cm^−3^), the S‐MgO/epoxy composite demonstrated a distinct advantage by achieving a higher thermal conductivity relative to its density. In addition, the 3D‐MgO/epoxy composite fabricated using the template method (51.94 vol%) exhibited a thermal conductivity of 6.61 W m^−1^ K^−1^.^[^ ^37^ ^]^ Despite having a filler volume fraction similar to that of the S‐MgO/epoxy composite, its thermal conductivity was significantly lower than that of the composite produced via the protein foaming method. This difference arises because the template method leads to an irregular filler‐particle distribution and inefficient particle connections, whereas the protein foaming method effectively forms dense and continuous networks. This structural advantage significantly enhances thermal conductivity, demonstrating that the protein foaming method is an effective approach for constructing dense and continuous 3D networks to achieve superior thermal performance. Our composite demonstrated superior thermal‐transport properties compared to most ceramic filler/epoxy composites, achieving higher thermal conductivity with lower filler loading. Notably, the S‐MgO/epoxy composite (54.64 vol%) exhibited superior thermal conductivity compared to the composite with a higher filler loading. This study highlights the unique thermal‐transport network in our composites, created by the protein foaming method for dense, continuous networks and liquid‐phase sintering with additives to smooth filler surfaces. Consequently, the MgO‐based composite developed in this study fulfills the requirements of a simple manufacturing process, excellent thermal conductivity, low density, and cost‐effectiveness, making it a promising candidate for thermal‐management applications.

Although various filler alignment techniques have been developed for thermally conductive fillers, their application in MgO‐based composites has not been sufficiently explored. In particular, MgO has faced challenges in previous studies owing to its high moisture reactivity and the requirement for high‐temperature sintering, which has hindered its use as a filler.^[^ ^19^, ^20^, ^37^ ^]^ Consequently, comparative studies on the different fabrication methods for MgO composites are still limited. This study demonstrated that the construction of a segregated structure with MgO fillers, in which the limitations of conventional MgO were effectively overcome, led to a remarkable enhancement in thermal conductivity.

### 2.5 Thermal Stability, Dielectric, and Electrical Properties of S‐MgO

**Figure 6:**
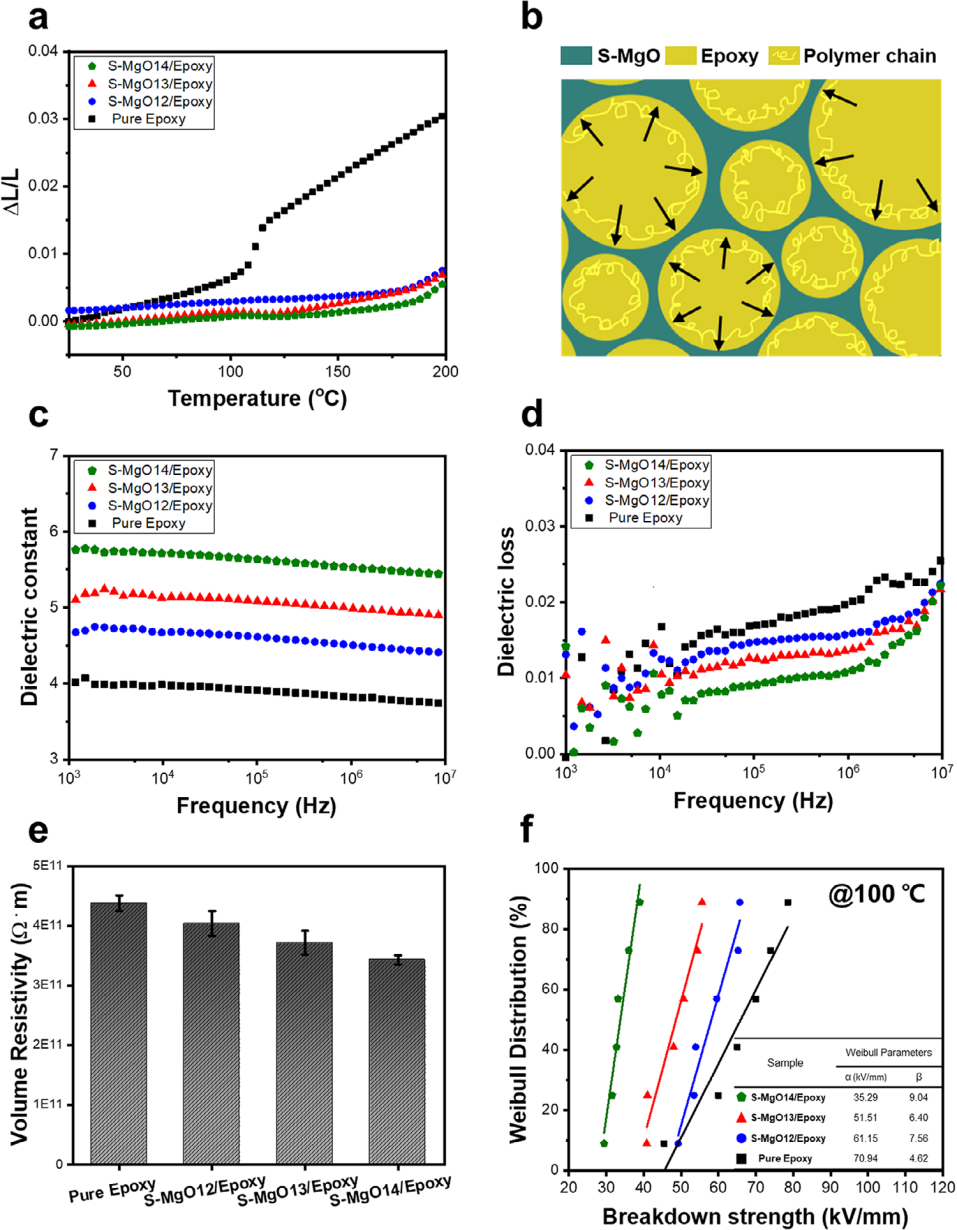
a) Characterization of CTE values, b) schematic diagram of thermal expansion stress, c) dielectric constant, d) dielectric loss tangent, e) volume resistivity, and f) Weibull distribution of breakdown strength for S‐MgO/epoxy composites under different sintering temperature conditions.

The coefficient of thermal expansion (CTE) of composites is closely related to the reliability of electronic devices. An appropriate CTE value can relieve thermal residual stresses and effectively reduce the occurrence of microcracks in electronic devices. Considering the operating temperature of electrical appliances, two distinct temperature ranges were considered for individual examinations. In this study, the S‐MgO/epoxy composites were observed to effectively reduce the CTE within the operating temperature range of 25 to 100 °C compared to pure epoxy (**Figure** **6**a). For example, the S‐MgO13/epoxy composites, which exhibited high thermal conductivity, had a CTE of 27.76 ppm °C^−1^, which was approximately three times lower than that of pure epoxy (83.06 ppm °C^−1^). In addition, even under overheating conditions from 125 to 175 °C, the S‐MgO13/epoxy composites consistently maintained a CTE value as low as 57.06 ppm °C^−1^, demonstrating excellent thermal stability. The sintered 3D‐MgO framework induced a strong confinement effect on the movement of the epoxy molecular chains, thereby improving the thermal stability.^[^ ^2^ ^]^ As shown in Figure 6b, the cell walls of the S‐MgO framework also contribute to relieving internal stress, thereby ensuring the macroscopic stability of the samples. Furthermore, as the sintering temperature increased, the content of the framework increased and the structure strengthened, further restricting the mobility of the epoxy molecular chains. This phenomenon clearly indicates that the volume change of the composite was effectively suppressed, while maintaining excellent dimensional stability. Consequently, the S‐MgO framework was effective in enhancing the thermal stability of polymer‐based composites, even in high‐temperature environments.

The materials used in integrated circuits must exhibit low dielectric properties to optimize device performance. In particular, a low dielectric constant is crucial for electronic‐packaging materials to achieve high‐speed operation, low dynamic power consumption, and reduced crosstalk noise.^[^ ^2^, ^3^, ^47^ ^]^ Figure 6c,d presents the dielectric properties of S‐MgO/epoxy composites with increasing sintering temperature, measured over a frequency range of 10^3^–10^7^ Hz at room temperature. The dielectric‐constant spectra of all samples remained relatively stable over the entire frequency range, indicating the excellent frequency independence of the materials (Figure 6c). Additionally, as the sintering temperature increased, a slight increase in the dielectric constant was observed. The S‐MgO13/epoxy composite exhibited a relatively low dielectric constant of ≈5.10 at 1 kHz, which was slightly higher than that of pure epoxy (4.02 at 1 kHz). This increase can be attributed to the higher dielectric constant of MgO compared with that of pure epoxy.^[^ ^37^ ^]^ Furthermore, the dielectric loss of the composites decreased with increasing sintering temperature, showing a lower dielectric loss than that of pure epoxy (Figure 6d). For example, the dielectric‐loss tangent of the S‐MgO13/epoxy composite at 10^7^ Hz was 0.022, which was lower than that of pure epoxy (0.026). The dielectric‐loss tangent for all composites remained below 0.03 over a wide frequency range, meeting the requirements for low dielectric loss in electronic devices. The reduction in the dielectric loss of the composite can be attributed to the intrinsically low dielectric loss of MgO and formation of a 3D network structure by the MgO fillers.^[^ ^53^ ^]^ Maxwell–Wagner–Sillars (MWS) polarization refers to the accumulation of charges at the interfaces between two materials with different dielectric constants and electrical conductivities, which induces interfacial polarization and contributes significantly to the increase in dielectric loss, particularly in composite materials such as ceramic filler/polymer systems.^[^ ^53^, ^54^ ^]^ In randomly dispersed composites, isolated interfaces promote more effective interfacial polarization. However, in the interconnected 3D structure formed by sintered MgO fillers, continuous interfacial networks are established, minimizing the number of interfaces between the fillers and the epoxy matrix. This reduces the total interfacial area and limits effective MWS polarization under an applied electric field.^[^ ^54^ ^]^ The enhanced interfacial connectivity reduces the number of isolated interfaces while simultaneously suppressing efficient polarization and minimizing overall dielectric loss. As a result, the 3D‐structured composites exhibit a decrease in dielectric loss and consequently improve the overall dielectric performance of the composite.

The insulating properties of the S‐MgO/epoxy composites were assessed by measuring their volume resistivities and breakdown strengths; the results are presented in Figure 6e,f. The volume resistivity of all composites was maintained at a high value of above 3 × 10^11^ Ω m, demonstrating excellent insulation characteristics. Additionally, as the sintering temperature of MgO increased, the volume resistivity decreased.^[^ ^8^, ^33^, ^37^ ^]^ This is attributed to the increased volume fraction of MgO, which exhibited superior electrical conductivity. Figure 6f shows the Weibull distribution of breakdown strength for each specimen. In the Weibull distribution, β is the shape parameter reflecting the data distribution, and α represents the characteristic breakdown strength at a 63.2% cumulative‐failure probability. As shown in Figure 6f, the breakdown strength of pure epoxy was measured at 70.94 kV mm^−1^. An increase in the filler volume fraction generally leads to a decrease in the breakdown strength of polymer composites.^[^ ^4^, ^5^ ^]^ The results indicated that the breakdown strength decreased as the volume fraction of MgO increased. The breakdown strengths of the S‐MgO12/epoxy and S‐MgO13/epoxy composites were 61.15 and 51.51 kV mm^−1^, respectively, which are slightly lower than that of pure epoxy. However, for the S‐MgO14/epoxy composite, the breakdown strength decreased to 35.29 kV mm^−1^, approximately two times lower than that of the pure epoxy, which can be attributed to the influence of pores.^[^ ^5^ ^]^

These findings suggest that MgO‐based composites not only achieve high thermal conductivity through a simple fabrication process, but also ensure excellent thermal stability, desirable dielectric properties, and electrical insulation. Furthermore, they highlighted the potential of MgO as a competitive material for thermal‐management applications owing to its low density and cost‐effectiveness. Future research should focus on strategies for reducing the interfacial thermal resistance and enhancing the thermal conductivity of MgO‐based TIMs. Accordingly, surface‐modification techniques should be actively investigated to enhance the hydrophobicity of MgO and improve its compatibility with polymer matrices.^[^ ^55^ ^]^ Furthermore, the adoption of advanced strategies, such as the design of 3D network structures and development of hybrid TIMs, is required to optimize the heat‐transfer pathways while ensuring scalability.^[^ ^55^, ^56^ ^]^ This approach is expected to accelerate the commercialization of MgO‐based composites and strengthen their competitiveness as high‐performance thermal‐management materials.

## 3 Conclusion

In this study, a 3D segregated MgO structure was successfully fabricated using a simple and eco‐friendly protein foaming method. This approach effectively aligned the filler particles within the 3D structure to form continuous and efficient thermal‐conduction pathways, demonstrating its ability to achieve superior thermal conductivity. The addition of TiO_2_ and Nb_2_O_5_ sintering additives to MgO effectively reduced the sintering temperature, leading to liquid‐phase sintering and the formation of a smooth surface layer with a thickness of a few nanometers. The contact angle of this surface layer with epoxy was measured to be 35.49°, compared to 105.81° for pure MgO, confirming its enhanced interfacial compatibility with epoxy. In addition, the sintering process effectively connected the particles within the framework, thereby minimizing the thermal resistance and maximizing the thermal conductivity of the composite. As a result, thermal‐conductivity measurements revealed that the S‐MgO/epoxy composite, with an MgO content of 54.64 vol%, exhibited an exceptionally high thermal conductivity of 17.19 W m^−1^ K^−1^. This value was 101 times higher than that of pure epoxy (0.17 W m^−1^ K^−1^) and approximately 3.7 times higher than that of a randomly dispersed MgO/epoxy composite (4.63 W m^−1^ K^−1^). Notably, the thermal conductivity of the composite exceeded that of expensive nitride‐based composites, demonstrating the potential of MgO‐based materials as cost‐effective alternatives. These results suggest that the outstanding thermal conductivity of MgO and the 3D segregated structure significantly enhanced the thermal conductivity of the composite. Furthermore, the composite exhibited excellent thermal stability, characterized by a low CTE of 27.76 ppm °C^−1^, as well as outstanding electrical‐insulation properties, including a low dielectric constant of 5.10 and high dielectric breakdown strength of 51.51 kV mm^−1^. These findings indicated that the 3D segregated structure of MgO positively affected the thermal and electrical properties of the composite. Considering these advantages, S‐MgO/epoxy composites are promising candidates for use as thermal‐interface materials in applications requiring both high thermal conductivity and electrical insulation.

## 4 Experimental Section

### Materials

MgO (purity > 99.9%, High Purity Chemicals, Japan), TiO_2_ (purity > 99.9%, Sigma Aldrich, USA), and Nb_2_O_5_ (purity > 99.9%, Sigma‐Aldrich, USA) powders were mixed by ball‐milling to prepared MgO + 0.15 at% TiO_2_ + 0.10 at% Nb_2_O_5_.^[^ ^20^ ^]^ The epoxy solution was prepared from the diglycidyl ether of bisphenol‐A (DGEBA, YD‐128, Kukdo Chemical, Republic of Korea), methyl tetrahydrophthalic anhydride (MTHPA, Kukdo Chemical, Republic of Korea), and *N*,*N*‐dimethylbenzylamine (BDMA, Sigma‐Aldrich, USA) at a weight ratio of 100:90:0.5.

### Preparation of the MgO Ceramic Fillers/Epoxy Composites

This study utilized an egg albumen‐assisted synthesis method. Albumen was extracted from fresh eggs after removing the yolk and chalaza. The albumen was then whipped in a beaker using a shear mixer (T25 digital ULTRA TURRAX, IKA, Germany). After 5 min, white sugar was gradually added to the albumen at a weight ratio of 1:1, and the mixture was whipped for an additional 15 min. until the sugar was completely dissolved, resulting in a porous albumen foam. Subsequently, MgO powder with additives was incorporated into the albumen at a volume fraction of 50 vol% to form a slurry. The slurry was then poured into a cylindrical silicone mold (30 mm in diameter and 1 cm in thickness) coated with silicone oil to facilitate easy demolding. The slurry was subjected to thermal consolidation in a convection oven at 80 °C for 24 h, allowing the protein to gradually solidify and ensuring the foam structure remained stable. Thereafter, the proteins were removed by burnout at a temperature of 500 °C for 2 h, then sintered at 1200, 1300, and 1400 °C for 2 h in air. The freestanding S‐MgO skeletons were obtained and placed in a Teflon mold. The epoxy was then poured into the Teflon mold, and the S‐MgO structure was infiltrated with an epoxy solution overnight under vacuum conditions. Finally, S‐MgO/epoxy composites were cured at 150 °C for 2 h in a drying oven.

The randomly dispersed MgO (R‐MgO)/epoxy composites were prepared by mixing size‐classified MgO fillers sintered at temperatures below 1500 °C (ExiAl 100, 60, and 20, Soulmaterial, Republic of Korea) with an epoxy resin through a simple mixing process. The ceramic fillers (100, 60, 20 µm) were mixed in a 100:60:20 = 2:1:1 ratio. The MgO fillers were mixed with epoxy resin at a volume ratio of 54.64:45.36, corresponding to an MgO volume fraction of 54.64 vol% obtained from the TGA analysis of S‐MgO13. The MgO filler and epoxy resin were mixed using a planetary mixer (PDM‐300, KM Tech, Republic of Korea) at 2000 rpm for 6 min. After mixing, the slurry was placed in a stainless‐steel mold (diameter: 12.7 mm), and then compressed and cured using an electric hot‐press machine (973214A, Carver, USA) at 160 °C under 50 MPa for 10 min. Finally, R‐MgO/epoxy composites were cured at 150 °C for 2 h in a drying oven.

### Characterizations

The microstructures of the 3D segregated MgO/epoxy composites were observed using a field emission scanning electron microscope (JSM‐7001 F, JEOL Co. Ltd., Japan) operated at 15 kV and EDS images were obtained using SEM. Micro‐CT (XT H 160, Nikon Instruments, Japan) was used to evaluate the 3D internal structure of the skeleton. In addition, the wettabilities of pure MgO and S‐MgO with the epoxy polymer were evaluated via contact‐angle measurements using a contact‐angle goniometer (Phoenix‐MT (A), SEO). The filler loading volume of MgO in the 3D segregated MgO/epoxy composites was precisely estimated using TGA (STA 449 F5_Jupiter, NETZSCH, Germany), with the samples heated from 100 to 700 °C at a heating rate of 20 °C min^−1^ under a nitrogen atmosphere. Error bars are not applicable for this measurement, as the presented data represent a qualitative assessment. Mercury porosimetry (Autopore V 9620, Micromeritics, USA) was used to characterize the porosity and average pore diameter. The chemical structures are analyzed using by XRD (D/max 2500, Rigaku, Japan) with Cu Kα radiation at 1.54056 Å. The thermal conductivities (*K*) of the composites were calculated based on the relationship *K* = *α*·*ρ*·*C*_p_, where *α* corresponds to the thermal diffusivity measured by LFA (LFA 467, NETZSCH, Germany) at ambient temperature, *ρ* is the density of the composites determined by Archimedes method, and *C*_p_ is the heat capacity of the composites tested using differential scanning calorimety.^[^ ^57^ ^]^ To ensure the accuracy and cross‐validation of the results, the thermal conductivity was also measured using the ASTM D 5470 steady‐state method (Dyntim S., Mentor Graphics, Republic of Korea), which is widely adopted in industry to evaluate the thermal conductivity of thermal interface materials. The TCE was calculated based on the average thermal conductivity values measured for each sample, relative to the thermal conductivity of pure epoxy. Since TCE is a derived value based on averaged data, error bars are not applicable. The heat transport properties of the specimens were characterized using an infrared thermal imaging camera (Xi 400 cam, Optris, Germany) with an infrared (IR) resolution of 382 × 288 pixels. All specimens were simultaneously placed on an aluminum plate preheated to 70 °C, and their temperature variations over time were monitored using an IR camera. The CTE of the composites were determined using thermomechanical analysis (Q400, TA Instruments, USA). Specimens with dimensions of 5 mm × 5 mm × 2 mm were measured in single cantilever bending mode at 1 Hz, within a temperature range of 25–200 °C a heating rate of 5 °C min^−1^. The dielectric constant and dielectric loss were measured using an impedance analyzer (E4990A, Keysight, USA) at 25 °C from 10^3^ to 10^7^ Hz and the volume resistivity was analyzed using an electrometer (6517B, Keithley, USA). The breakdown strengths were measured with a DC ramp voltage of 100 V s^−1^ using a high voltage generator (HPM‐W30SA, Han‐Tech, Republic of Korea) as the voltage source in silicone oil, which can withstand high temperatures and was equipped with a thermocouple for temperature control.

## Conflict of Interest

The authors declare no conflict of interest.

## Supporting information



Supporting Information

MovieS1

## Data Availability

The data that support the findings of this study are available from the corresponding author upon reasonable request.
