# Supplementary material for: Simple Protein Foaming‐Derived 3D Segregated MgO Networks in Epoxy Composites with Outstanding Thermal Conductivity Properties
Source: Adv Sci (Weinh). 2025 May 28;12(33):e06465. doi: 10.1002/advs.202506465 (PMC12412586; doi:10.1002/advs.202506465)
Supplement: Supplementary file 1 — Supporting Information [file ADVS-12-e06465-s002.docx]

Supporting Information

**Simple Protein Foaming-Derived Three-Dimensional Segregated MgO Networks in Epoxy Composites with Outstanding Thermal Conductivity Properties**

*Su-Jin Ha, Young Kook Moon, Jong‑Jin Choi, Byung‑Dong Hahn, Cheol‑Woo Ahn, Kyung-Hoon Cho*, Hyun-Ae Cha***

S.-J. Ha, Y. K. Moon, J.-J. Choi, B.-D. Hahn, C.-W. Ahn, Hyun-Ae Cha

Nano Materials Research Division Korea Institute of Materials Science (KIMS), Changwon, Gyeongnam 641-831, Republic of Korea

E-mail: *hacha@kims.re.kr*

S. J. Ha, K. H. Cho

School of Materials Science and Engineering, Kumoh National Institute of Technology, 61 Daehak-ro, Gumi, Gyeongbuk 39177, Republic of Korea

*E-mail: khcho@kumoh.ac.kr*


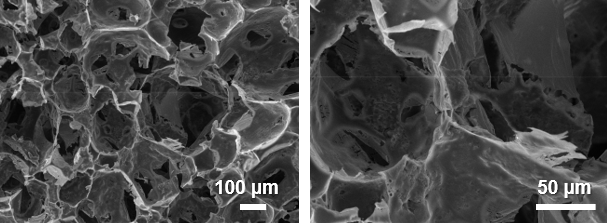


**Figure S1.** SEM images of the protein foaming-derived hierarchical porous albumen.

**
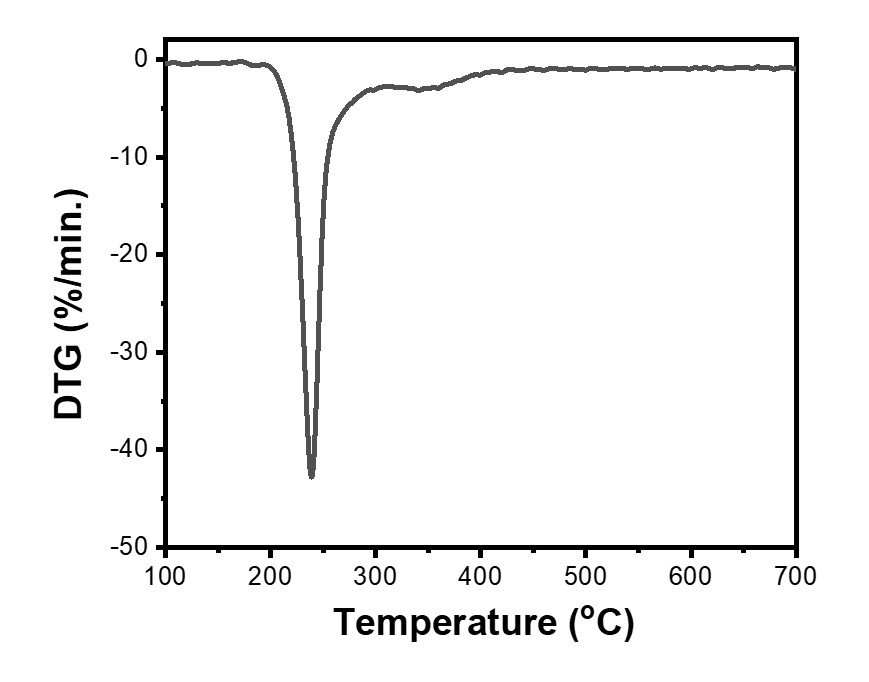
**

**Figure S2.** Derivative curves of the protein foaming-derived hierarchical porous albumen.


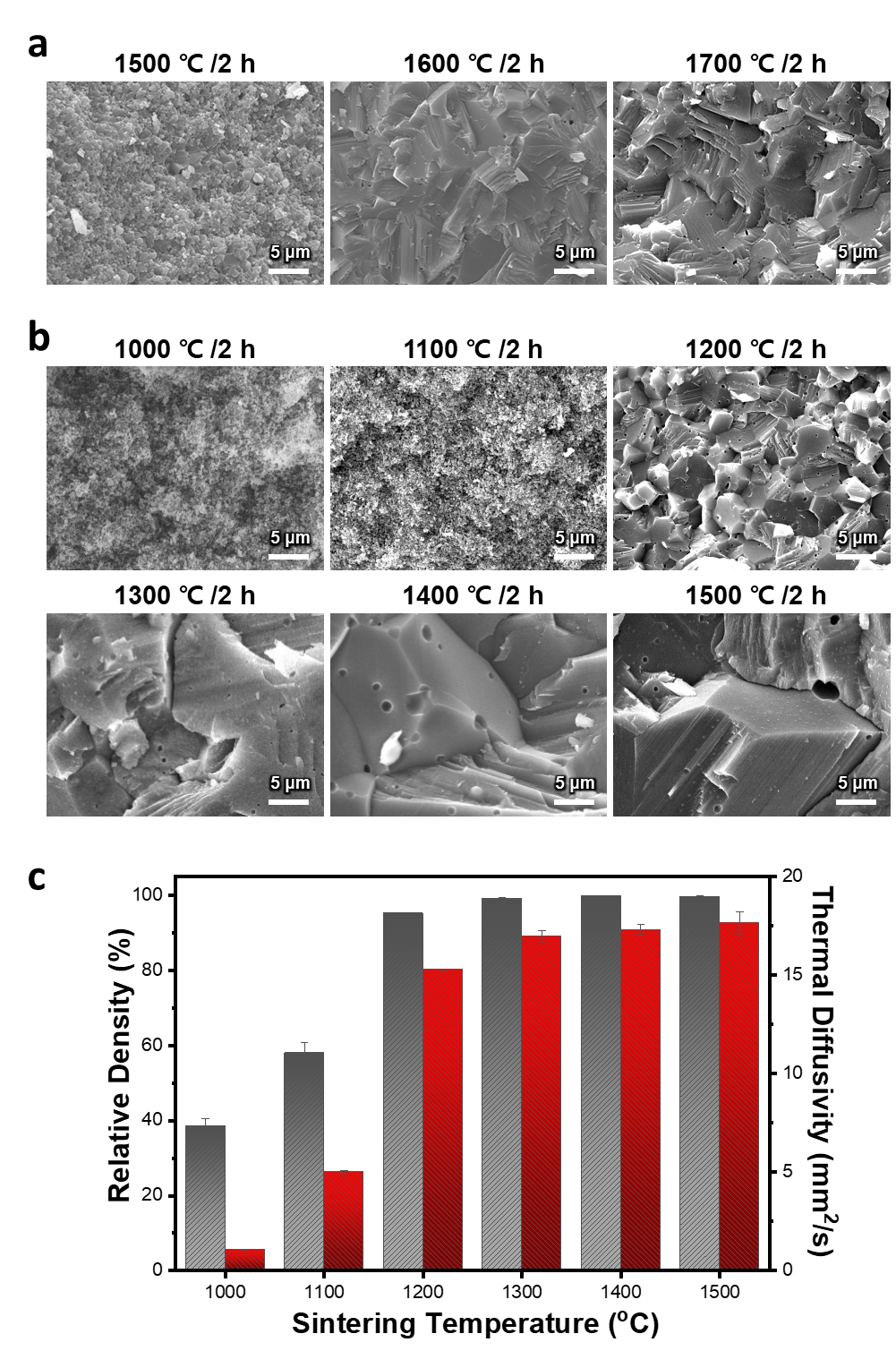


**Figure S3.** a) SEM images of sintered pure MgO ceramics at temperatures ranging from 1500 to 1700 °C for 2 h. b) SEM images, and c) relative density and thermal diffusivity of sintered MgO ceramics with TiO_2_ and Nb_2_O_5_ additives at temperatures ranging from 1000 to 1500 °C for 2 h.

**
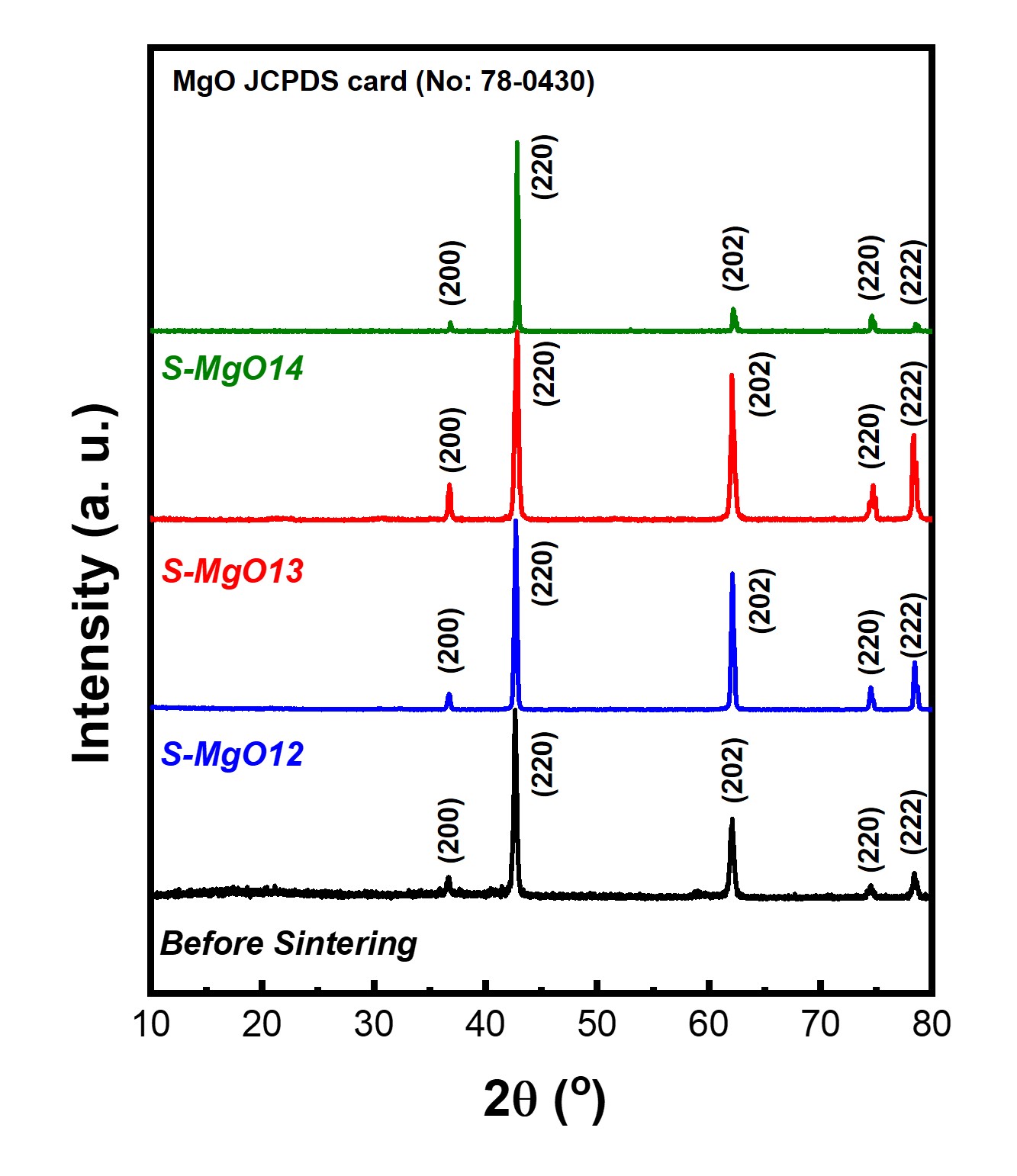
**

**Figure S4.** XRD patterns of protein foaming derived S-MgO frameworks before and after sintering at each sintering temperature.


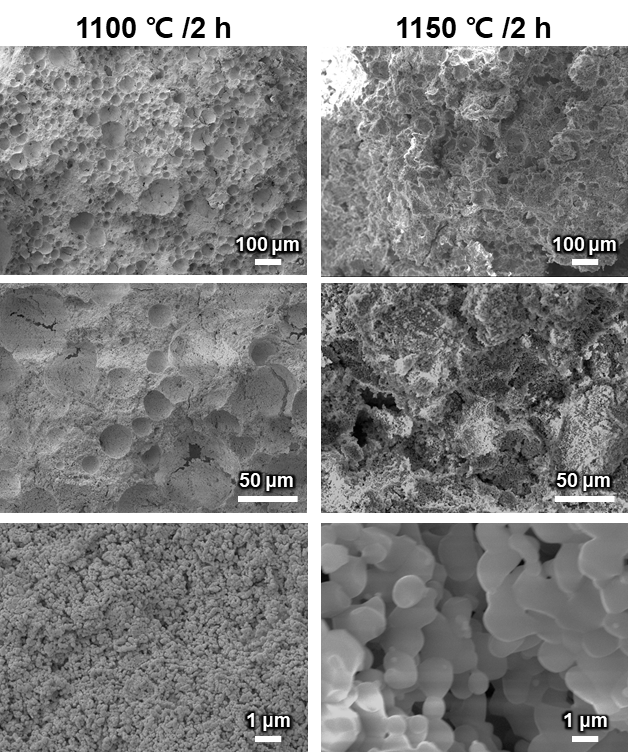


**Figure S5.** SEM images of S-MgO framework after sintering at 1000 °C and 1150 °C for 2 h.


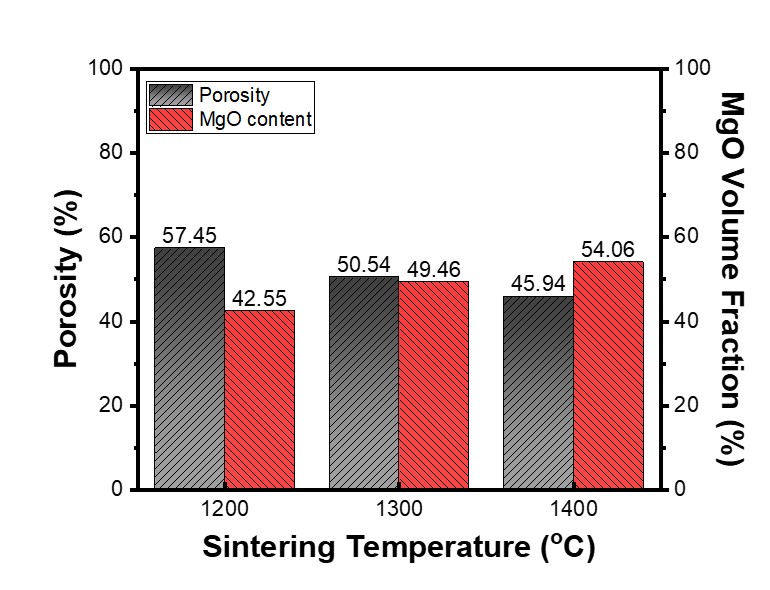


**Figure S6.** Porosity and MgO volume fraction of S-MgO framework via porosimetry.

**
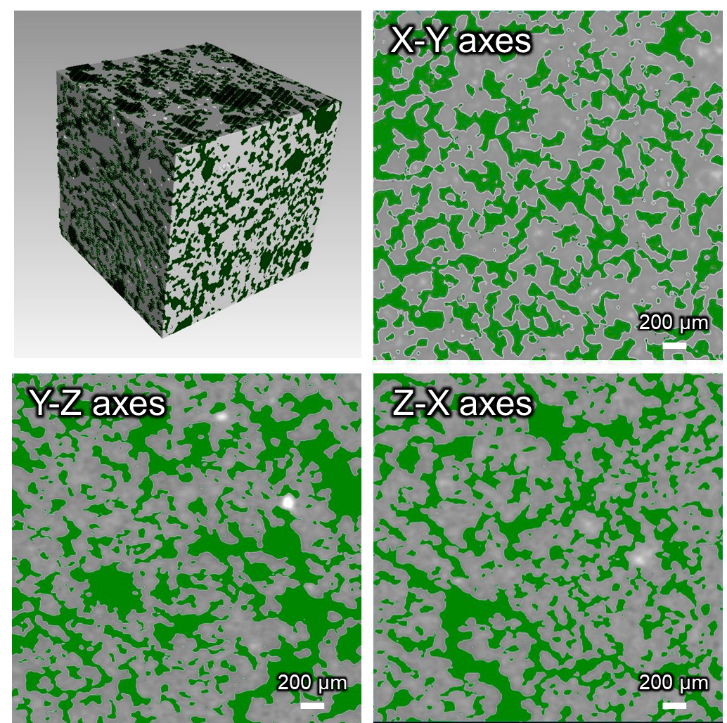
**

**Figure S7.** Micro-CT images of X-Y, Y-Z, and Z-X axes of S-MgO13.


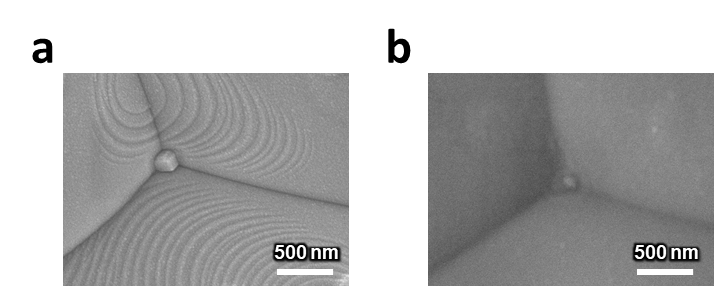


**Figure S8.** The SEM images of the surfaces of a) pure MgO sintered at 1700 ^o^C and b) MgO with additives sintered at 1300 ^o^C.


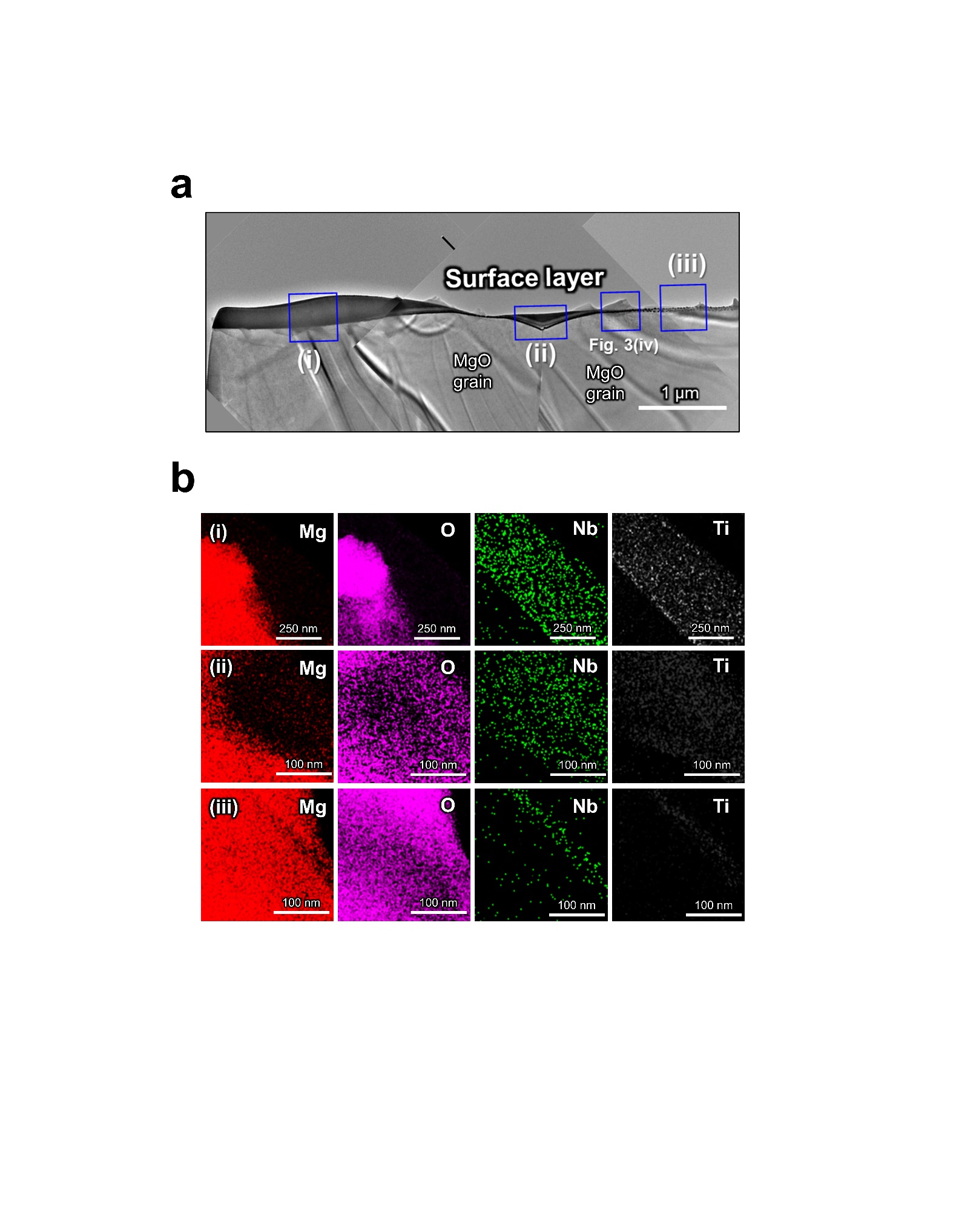


**Figure S9.** a) TEM image and b) EDS analysis of MgO with additives.

**
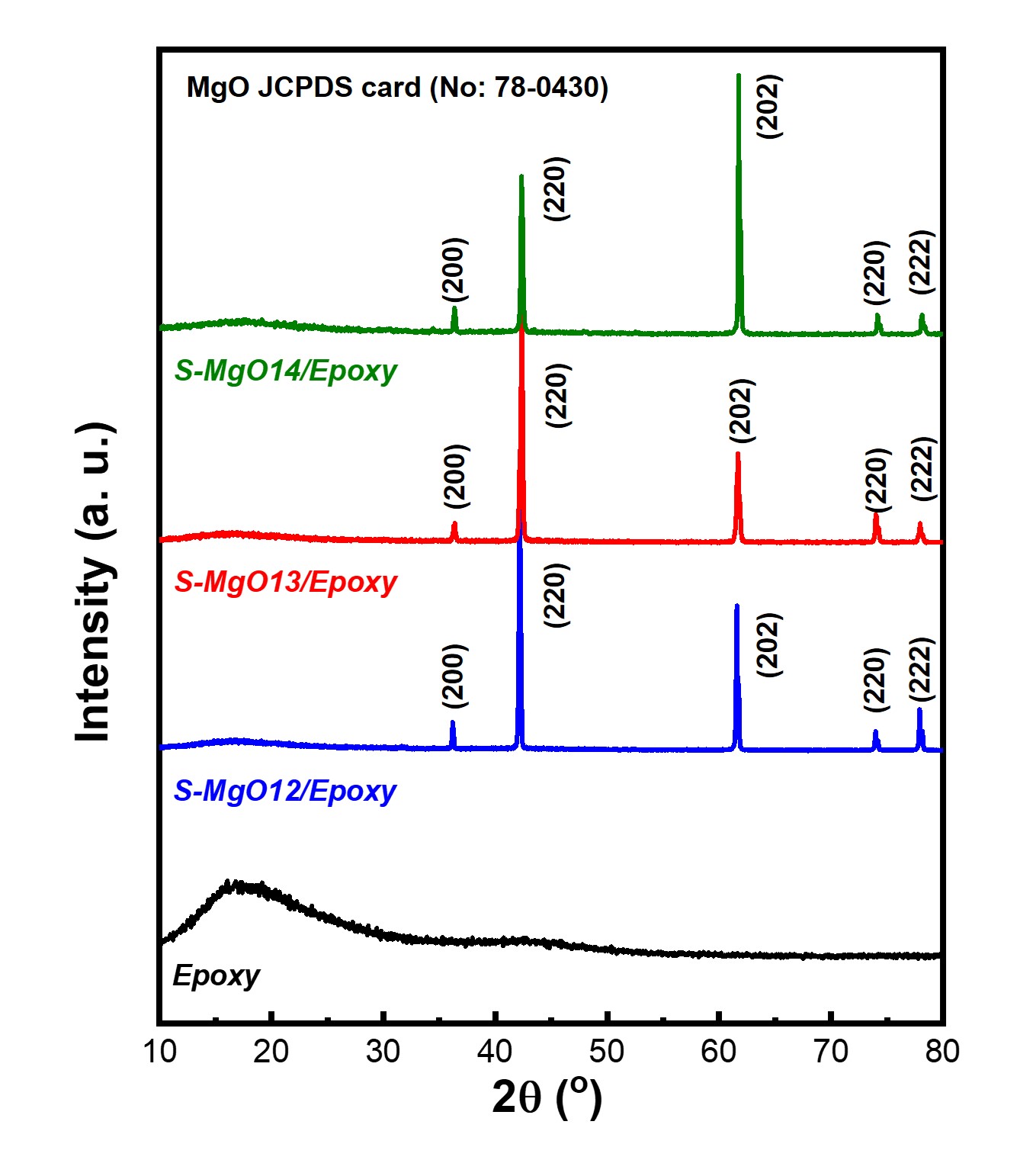
**

**Figure S10.** XRD patterns of pure epoxy and S-MgO/epoxy composites sintered at each sintering temperature.


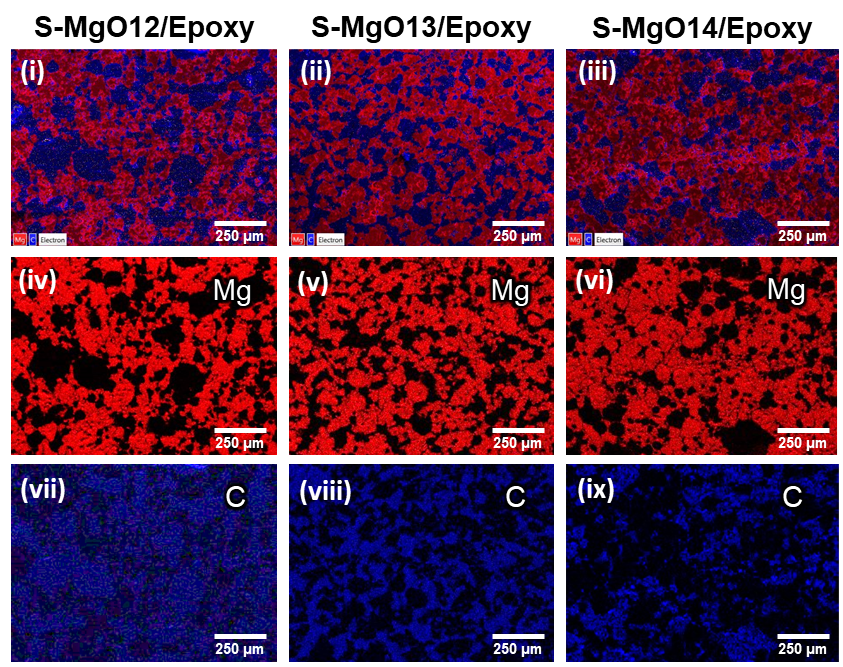


**Figure S11.** The EDS elemental mapping results of S-MgO/epoxy composites with 3D segregated framework sintered at each sintering temperature.

**
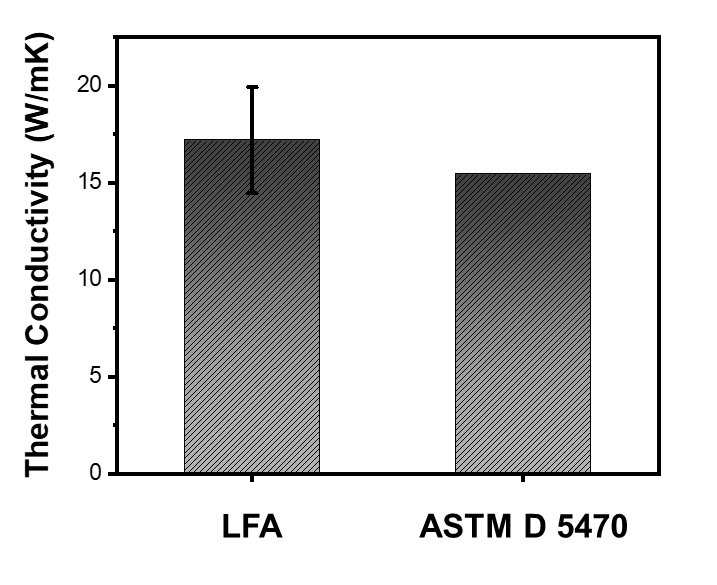
**

**Figure S12.** Thermal conductivity comparison of the S-MgO13/epoxy composite measured using LFA and ASTM D 5470 methods.


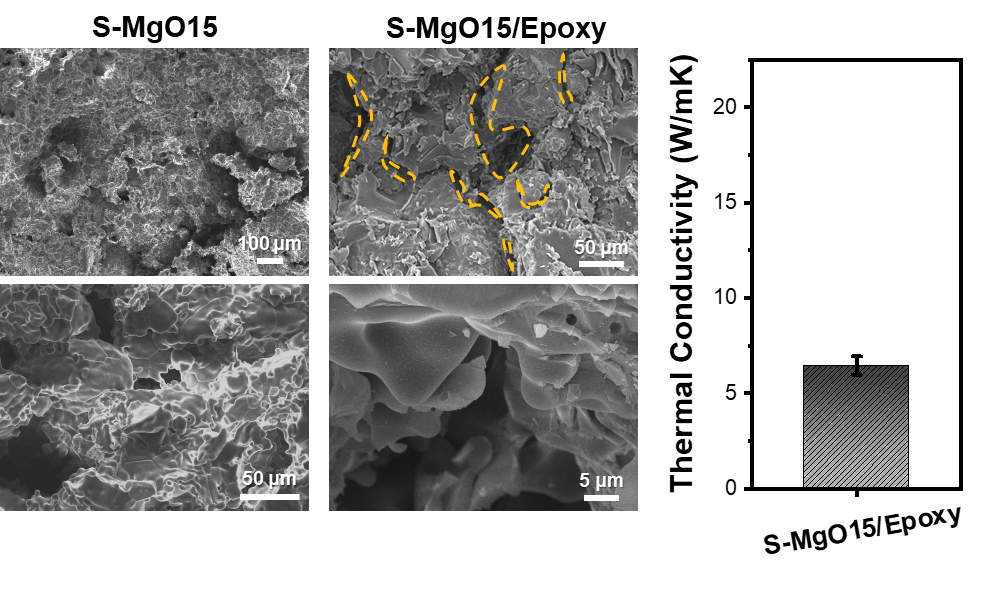


**Figure S13.** SEM images of S-MgO15 and S-MgO15/epoxy composites at 1500 °C and thermal conductivity of S-MgO15/epoxy composite.


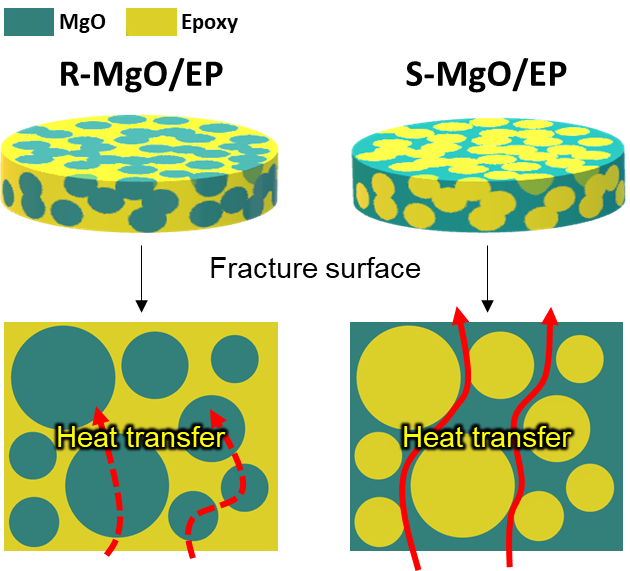


**Figure S14.** Schematic diagram of thermal transport in R-MgO and S-MgO.

**
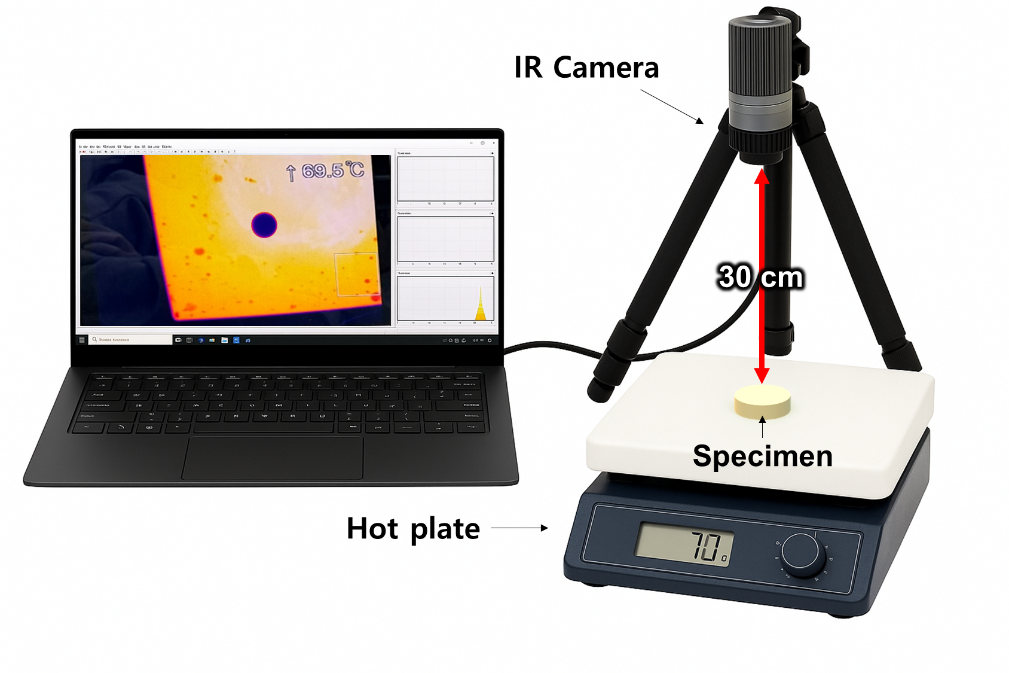
**

**Figure S15.** Schematic illustration of the experimental setup for infrared thermal imaging measurements.

**Table S1.** Thermal conductivity and density of various ceramic filler/epoxy composites reported.

| Random-structure | | | | | |
| --- | --- | --- | --- | --- | --- |
| **Composite Materials** | | **Filler Volume (%)** | **Thermal Conductivity (W/mK)** | **Density (g/cm^3^)** | **Reference #** |
| Al_2_O_3_/EP | | 58.4 | 3.40 | 2.81 | ^[3]^ |
| Al_2_O_3_/EP | | 43 | 4.36 | 2.38 | ^[4]^ |
| Al_2_O_3_/EP | | 60 | 4.30 | 2.85 | ^[5]^ |
| Al_2_O_3_/EP | | 41.4 | 1.07 | 2.34 | ^[6]^ |
| Al_2_O_3_/EP | | 53.8 | 4.27 | 2.62 | ^[7]^ |
| Al@Al_2_O_3_/EP | | 31.3 | 0.92 | 2.06 | ^[8]^ |
| Al_2_O_3_-0.3%MWCNT/EP | | 49.1 | 2.60 | 2.55 | ^[9]^ |
| AlN/EP | | 57 | 3.39 | 2.37 | ^[10]^ |
| AlN/GO/EP | | 33 | 2.77 | 1.83 | ^[11]^ |
| BN/EP | | 44 | 9.00 | 1.66 | ^[12]^ |
| BN/EP | | 25 | 3.58 | 1.44 | ^[14]^ |
| Si_3_N_4_/EP | | 62.5 | 1.70 | 2.45 | ^[15]^ |
| Si_3_N_4_/EP | | 60 | 3.00 | 2.4 | ^[16]^ |
| Si_3_N_4_/EP | | 60 | 2.60 | 2.4 | ^[17]^ |
| Si_3_N_4_/EP | | 45 | 1.75 | 2.1 | ^[18]^ |
| MgO/EP | | 90 | 7.50 | 3.34 | ^[20]^ |
| MgO/EP | | 44 | 3.16 | 2.25 | ^[21]^ |
| MgO/EP | | 33 | 1.41 | 1.99 | ^[22]^ |
| 3D-structure | | | | | |
| **Composite Materials** | **Method** | **Filler Volume (%)** | **Thermal Conductivity (W/mK)** | **Density (g/cm^3^)** | **Reference #** |
| 3D-Al_2_O_3_/EP | Gel-casting | 70 | 13.46 | 3.13 | ^[26]^ |
| 3D-Al_2_O_3_/EP | Pore foaming | 31.3 | 4.10 | 2.06 | ^[29]^ |
| 3D-α-Al_2_O_3_/EP | Protein foaming | 23.32 | 2.58 | 1.84 | ^[2]^ |
| 3D-Al_2_O_3_/Fe_3_O_4_/EP | Magnetic fields | 34.63 | 2.30 | 2.15 | ^[41]^ |
| 3D-AlN/EP | Pore foaming | 39.69 | 4.29 | 2.02 | ^[30]^ |
| 3D-AlN/EP | Pore foaming | 20 | 2.26 | 1.61 | ^[31]^ |
| 3D-AlN/Fe_3_O_4_/EP | Magnetic fields | 20 | 1.75 | 1.61 | ^[42]^ |
| 3D-BN/EP | Direct foaming | 19.40 | 1.62 | 1.53 | ^[27]^ |
| 3D-BN/EP | Sacrificial template | 80 | 13.65 | 2.07 | ^[32]^ |
| 3D-BN/EP | Sacrificial template | 19.8 | 2.54 | 1.38 | ^[33]^ |
| 3D-BN/EP | Sacrificial template | 14.61 | 2.11 | 1.36 | ^[34]^ |
| 3D-BN/EP | Sacrificial template | 13.16 | 5.05 | 1.30 | ^[35]^ |
| 3D-BN/Al_2_O_3_/EP | Sacrificial template | 13.61 | 1.08 | 1.57 | ^[36]^ |
| 3D-BN/Fe_3_O_4_/EP | Magnetic field | 62.6 | 12.10 | 1.3 | ^[43]^ |
| 3D-Si_3_N_4_/EP | Direct foaming | 41 | 2.99 | 2.01 | ^[28]^ |
| 3D-Si_3_N_4_/EP | Protein foaming | 22.2 | 3.89 | 1.64 | ^[52]^ |
| 3D-MgO/EP | Sacrificial template | 51.94 | 6.61 | 2.44 | ^[37]^ |
| **This work** | Protein foaming | **54.6** | **17.19** | **2.48** |  |
